# On the convergence of the gradient projection method for convex optimal control problems with bang–bang solutions

**DOI:** 10.1007/s10589-018-9981-6

**Published:** 2018-01-29

**Authors:** J. Preininger, P. T. Vuong

**Affiliations:** Institute of Statistics and Mathematical Methods in Economics, Vienna University of Technology, Vienna, Austria

**Keywords:** Gradient projection method, Strong convergence, Convergence rate, Optimal control, Bang–bang control, 47J20, 49J15, 49M05, 90C25, 90C30

## Abstract

We revisit the gradient projection method in the framework of nonlinear optimal control problems with bang–bang solutions. We obtain the strong convergence of the iterative sequence of controls and the corresponding trajectories. Moreover, we establish a convergence rate, depending on a constant appearing in the corresponding switching function and prove that this convergence rate estimate is sharp. Some numerical illustrations are reported confirming the theoretical results.

## Introduction

Numerical solution methods for various optimal control problems have been investigated during the last decades [6, 8–11]. However, in most of the literature, the optimal controls are assumed to be at least Lipschitz continuous. This assumption is rather strong, as whenever the control appears linearly in the problem, the lack of coercivity typically leads to discontinuities of the optimal controls. Recently, optimal control problems with bang–bang solutions attract more attention. Stability and error analysis of bang–bang controls can be found in [14, 26, 32]. Euler discretizations for linear–quadratic optimal control problems with bang–bang solutions were studied in [1, 2, 5, 29]. Higher order schemes for linear and linear–quadratic optimal control problems with bang–bang solutions were developed in [24, 27].

On the other hand, among many traditional solution methods in optimization, projection-type methods are widely applied because of their simplicity and efficiency [13, 15, 31].

Recently, the gradient projection method has been reconsidered for solving general optimal control problems [22, 28]. Under some suitable conditions, it was proved that the control sequence converges weakly to an optimal control and the corresponding trajectory sequence converges strongly to an optimal trajectory. However, no convergence rate result has been established.

In this paper, we study the gradient projection method for optimal control problems with bang–bang solutions. In particular we consider the following problem

1.1 $$\begin{aligned} \text{ minimize } \psi (x,u):=g(x(T))+\int _0^T h(t,x(t),u(t))dt \end{aligned}$$

subject to

1.2 $$\begin{aligned} \dot{x}(t)=f(t,x(t),u(t))\quad \text{ for } \text{ a.e. }~ t\in [0,T], \quad x(0)=x_0, \end{aligned}$$

and

1.3 $$\begin{aligned} u(t)\in U:=[-1,1]^m\quad \text{ for } \text{ a.e. }~ t\in [0,T]. \end{aligned}$$

Here [0, *T*] is a fixed time horizon, admissible controls are all measurable functions $u:[0,T]\rightarrow U$, while $x(t)\in {\mathbb {R}}^n$ denotes the state of the system at time $t\in [0,T]$ and the functions $f:{\mathbb {R}}\times {\mathbb {R}}^n\times {\mathbb {R}}^m\rightarrow {\mathbb {R}}^n, g:{\mathbb {R}}^n\rightarrow {\mathbb {R}}$ and $h:{\mathbb {R}}\times {\mathbb {R}}^n\times {\mathbb {R}}^m\rightarrow {\mathbb {R}}$ are given.

Further we assume (see the next section for precise formulations) that the data are smooth enough, that the problem (1.1)–(1.3) is convex and that for the (unique) optimal control $u^*$ the objective function fulfills a certain growth condition. In particular we show that this condition is satisfied in the bang–bang case if each component of the associated switching function satisfies a growth condition as given in [25, 29].

Under these assumptions, we prove that the control sequence actually converges strongly to the solution. Moreover, the convergence rates for both controls and states are provided, depending on the constant appearing in the growth condition for the switching function. An example is analysed showing that the estimation for these convergence rates is sharp.

The paper is organized as follows: In Sect. 2, we specify the assumptions we use and recall some facts which will be useful in the sequel. Section 3 discusses the convergence properties of the gradient projection method. Some numerical examples of linear–quadratic type are reported in Sect. 4 illustrating the results in the previous section. Some final remarks are given in the last section.

## Preliminaries

In this section, we will clarify the assumptions used and recall some important facts which are necessary to establish our result.

By ${\mathcal {U}}:=L^2([0,T],U)$ we denote the set of all admissible controls and if not stated otherwise $\Vert \cdot \Vert$ denotes the $L^2$-norm. The first two assumptions guarantee that the problem (1.1)–(1.3) is meaningful.

### Assumption A1

For any given control $u\in {\mathcal {U}}$ there is a unique solution $x=x(u)$ of (1.2) on [0, *T*].

### Assumption A2

The problem (1.1)–(1.3) has a solution $(x^*,u^*)$.

Now recall the Hamiltonian of (1.1)–(1.3) as

$$\begin{aligned} H(t,x,u,p)=\langle p, f(t,x,u)\rangle +h(t,x,u). \end{aligned}$$

Then by the Pontryagin maximum principle there is an absolutely continuous function $p^*$ such that $(x^*,u^*,p^*)$ solves the adjoint equation

2.1 $$\begin{aligned} \dot{p}(t)= & {} -H_x(t,x(t),u(t)\nonumber \\= & {} -f_x(t,x(t),u(t))^\top p(t)- h_x(t,x(t),u(t))^\top \quad \text{ for } \text{ a.e. }~t\in [0,T]\nonumber \\ p(T)= & {} \nabla g(x(T)), \end{aligned}$$

and for every $u\in U$

$$\begin{aligned} \langle H_u(t,x^*(t),u^*(t),p^*(t)),u-u^*(t)\rangle \ge 0\quad \text{ for } \text{ a.e. }~t\in [0,T]. \end{aligned}$$

We define $J:{\mathcal {U}}\rightarrow {\mathbb {R}}$ via $J(u):=\psi (x(u),u)$, where *x*(*u*) is the solution (1.2). Then we have the following useful formula for the gradient of *J* (see, e.g. [22, 31]).

2.2 $$\begin{aligned} \nabla J(u)(t)= & {} H_u(t,x(t),u(t),p(t))\nonumber \\= & {} f_u(t,x(t),u(t))^\top p(t)+ h_u(t,x(t),u(t))^\top , \end{aligned}$$

where *x* and *p* are the unique solution of (1.2) and (2.1) depending on $u\in {\mathcal {U}}$.

### Assumption A3

The objective function *J* is continuously differentiable on ${\mathcal {U}}$ with Lipschitz derivative.

We denote by *L* the Lipschitz modulus of the gradient $\nabla J$ of *J* and write $J^*:=J(u^*)$ for its optimal value. The following result is well known (see e.g. [23, Lemma 1.30]).

### Lemma 2.1

Suppose that A3 is fulfilled. Then for every $u,v \in {\mathcal {U}}$ the following estimation holds

$$\begin{aligned} J(v)-J(u)-\left\langle \nabla J(u), v-u \right\rangle \le \frac{L}{2}\Vert v-u\Vert ^2. \end{aligned}$$

Assumptions A1–A3 are common in optimal control. For example the following two Assumptions B1–B2 imply A1–A3 (cf. [22])

### Assumption B1

The functions *f* and *h* are of the form $f(t,x,u)=f_0(x)+f_1(x)u$ and $h(t,x,u)=h_0(x)+\langle h_1(x),u\rangle$ respectively, where $f_0:{\mathbb {R}}^n\rightarrow {\mathbb {R}}^n, f_1:{\mathbb {R}}^n\rightarrow {\mathbb {R}}^{n\times m}, h_0:{\mathbb {R}}^n\rightarrow {\mathbb {R}}$ and $h_1:{\mathbb {R}}^n\rightarrow {\mathbb {R}}^m$ are twice continuously differentiable.

### Assumption B2

There exists $c\ge 0$ such that for every $x\in {\mathbb {R}}^n$ and $u\in U$:

$$\begin{aligned} \langle x,f(t,x,u)\rangle \le c(1+|x|^2). \end{aligned}$$

Additionally we assume the following.

### Assumption A4

The objective function *J* is convex.

Note that if the set ${\mathcal {F}}$ of admissible pairs is convex this assumption is equivalent to the statement that the function $\psi$ is convex on ${\mathcal {F}}$. In particular this is the case if *f* is affine (i.e. *f* is of the form $f(t,x,u)=A(t)x+B(t)u+d(t)$) as in [25, 29].

Further we will assume a growth condition for *J* that is similar to (4.7) in [3].

### Assumption A5

For a solution $u^*$ of (1.1)–(1.3) there are constants $\beta >0$ and $\theta \ge 0$ such that for every $u\in {\mathcal {U}}$ we have

$$\begin{aligned} J(u)-J(u^*)\ge \beta \Vert u-u^*\Vert ^{2\theta +2}. \end{aligned}$$

Note that in particular A5 implies that the solution $u^*$ is unique.

### Remark 2.2

For coercive optimal control problems (in the sense of [12]) Assumptions A1–A4 are fulfilled as well as A5 for $\theta =0$. In these problems the objective function *J* however is even strongly convex and therefore one can apply known results (e.g. [21, Theorem 2.1.15]) directly to show linear convergence of the gradient projection method in this case.

In the following we will show that Assumption A5 is fulfilled for bang–bang controls with no singular arcs. We recall that in the case of bang–bang controls the function $\sigma ^*:=H_u(\cdot ,x^*,u^*,p^*)$ is called *switching function* corresponding to the triple $(x^*,u^*,p^*)$. For every $j\in \{1,\ldots ,m\}$ denote by $\sigma ^*_j$ its *j*-th component. The following assumption says that the switching function $\sigma ^*$ satisfies a growth condition around the switching points, which implies that $u^*$ is strictly bang–bang.

### Assumption B3

There exist real numbers $\theta ,\alpha ,\tau >0$ such that for all $j\in \{1,\ldots ,m\}$ and $s\in [0,T]$ with $\sigma ^*_j(s)=0$ we have

$$\begin{aligned} |\sigma ^*_j(t)|\ge \alpha |t-s|^\theta \quad \forall t\in [s-\tau ,s+\tau ]\cap [0,T]. \end{aligned}$$

Assumption B3 plays the main role in the study of regularity, stability and error analysis of discretization techniques for optimal control problems with bang–bang solutions. Many variations of this assumption are used in the literature about bang–bang controls. To our knowledge the first assumption of this type was introduced by Felgenhauer [14] for continuously differentiable switching functions with $\theta =1$ to study the stability of bang–bang controls. Alt et al. [1, 2, 4] used a slightly stronger version of B3 with $\theta =1$, that additionally excludes the endpoints 0 and *T* as zeros of the switching function, to investigate the error bound for Euler approximation of linear–quadratic optimal control problems with bang–bang solutions. Quincampoix and Veliov [26] used a rank condition which implies B3 (including cases where $\theta \ne 1$) to obtain the metric regularity and stability of Mayer problems for linear systems. Seydenschwanz [29], Preininger et al. [25], Pietrus, Scarinci and Veliov [24, 27] used this assumption in the study of metric (sub)-regularity, stability and error estimate for discretized schemes of linear–quadratic optimal control problems with bang–bang solutions.

To prove that B3 implies A5 we need the following lemma, which is a simplified version of [29, Lemma 1.3] (see also, [1, Lemma 4.1]).

### Lemma 2.3

Let Assumptions A1–A2 be fulfilled and let $u^*$ be a solution of (1.1)–(1.3) such that B3 is fulfilled for some $\theta > 0$. Then there exists constants $\beta >0$ such that for any feasible $u\in {\mathcal {U}}$ it holds

$$\begin{aligned} \int _0^T \sigma ^*(t)^T\left( u(t)-u^*(t)\right) dt \ge \beta \Vert u-u^*\Vert _1^{\theta +1}, \end{aligned}$$

where $\Vert \cdot \Vert _1$ is the $L^1$-norm.

### Proposition 2.4

Let Assumptions A1, A2 and A4 be fulfilled and let $u^*$ be a solution of (1.1)–(1.3) such that B3 is fulfilled. Then A5 holds.

### Proof

From Assumption A4 and (2.2) we obtain

2.3 $$\begin{aligned} J(u) - J(u^*) \ge \left\langle \nabla J(u^*), u-u^*\right\rangle =\int _0^T \sigma ^*(t)^T\left( u(t)-u^*(t)\right) dt. \end{aligned}$$

Since $\Vert \cdot \Vert ^2 \le C\Vert \cdot \Vert _1$ on ${\mathcal {U}}$ for some constant $C>0$, from Lemma 2.3 there exists $\beta >0$ such that

2.4 $$\begin{aligned} \int _0^T \sigma ^*(t)^T\left( u(t)-u^*(t)\right) dt\ge \beta \Vert u-u^*\Vert _1^{\theta +1} \ge \frac{\beta }{C^{\theta +1}} \Vert u-u^*\Vert ^{2\theta +2}. \end{aligned}$$

Combining (2.3) and (2.4) we obtain A5. $\square$

To define the gradient projection method in the next chapter we will need the following notion of a projection. For each $u\in {\mathcal {U}}$, there exists a unique point in ${\mathcal {U}}$ (see [17, p. 8]), denoted by $P_{{\mathcal {U}}}(u)$, such that

$$\begin{aligned} \Vert u-P_{{\mathcal {U}}}(u)\Vert \le \Vert u-v\Vert \quad \forall v\in {\mathcal {U}}. \end{aligned}$$

It is well known [17, Theorem 2.3] that the projection operator can be characterized by

2.5 $$\begin{aligned} \langle u-P_{{\mathcal {U}}}(u),v-P_{{\mathcal {U}}}(u) \rangle \le 0 \quad \forall v\in {\mathcal {U}}. \end{aligned}$$

Further to establish the convergence rate of the gradient projection method, we will need the following lemmas.

### Lemma 2.5

[18, Lemma 7.1] Let $\alpha >0$ and let $\{\delta _k\}_{k=0}^{\infty }$ and $\{s_k\}_{k=0}^{\infty }$ be two sequences of positive numbers satisfying the conditions

$$\begin{aligned} s_{k+1}(\delta _k s_{k+1}^{\alpha }+1)\le s_k \quad \forall k\in {\mathbb {N}}. \end{aligned}$$

Then there is a number $\gamma >0$ such that

$$\begin{aligned} s_k\le \left( s_0^{-\alpha }+\gamma \sum _{i=0}^{k-1}\min \{\delta _i,\delta _i^{\frac{\alpha }{\alpha +1}}\}\right) ^{-\frac{1}{\alpha }} \quad \forall k\in {\mathbb {N}}. \end{aligned}$$

In particular, we have $\lim _{k\rightarrow \infty }s_k=0$ whenever $\sum _{k=0}^{\infty }\delta _k=\infty .$

### Lemma 2.6

[7, Lemma 3.2] Let $\left\{ \alpha _{k}\right\} , \left\{ s_{k}\right\}$ be sequences in ${\mathbb {R}}_{+}$ satisfying

$$\begin{aligned} \sum _{i=0}^{\infty } \alpha _{k} s_k < \infty , \end{aligned}$$

the sequence $\left\{ \alpha _{k}\right\}$ is non-summable and the sequence $\left\{ s_{k}\right\}$ is decreasing. Then

$$\begin{aligned} s_k=o\left( \frac{1}{\sum _{i=0}^{k}\alpha _{i}} \right) , \end{aligned}$$

where the *o*-notation means that $s_k = o(1/t_k)$ if and only if $\lim _{k \rightarrow \infty } s_k t_k = 0$.

## Convergence analysis

We consider the following Gradient Projection Method (GPM):

**Algorithm GPM**

- **Step 0:** Choose a sequence $\{\lambda _k\}$ of positive real numbers and an initial control $u_0\in {\mathcal {U}}$. Set $k=0$.
- **Step 1:** Compute the gradient $\nabla J(u_k)(t):=f_u(t,x_k(t),u_k(t))^\top p_k(t)+ h_u(t,x_k(t),u_k(t))^\top$ by solving the following differential equations
  3.1 $$\begin{aligned} \dot{x}_k(t)= & {} f(t,x_k(t),u_k(t)), \quad x_k(0)=x_0;\nonumber \\ \dot{p}_k(t)= & {} -f_x(t,x_k(t),u_k(t))^\top p_k(t)- h_x(t,x_k(t),u_k(t))^\top , \nonumber \\ p_k(T)= & {} \nabla g(x_k(T)). \end{aligned}$$
- **Step 2:** Compute
  3.2 $$\begin{aligned} {u}_{k+1} = P_{\mathcal {U}}(u_k-\lambda _k \nabla J(u_k)). \end{aligned}$$
- **Step 3:** If $u_{k+1}=u_k$ then Stop. Otherwise replace *k* by $k+1$ and go to **Step 1**.

It is known (see e.g. [21, Theorem 2.1.14]) that for *J* continuously differentiable with Lipschitz derivative the gradient (projection) method has the convergence rate $O(\frac{1}{k})$ in terms of the objective value. I.e. that

3.3 $$\begin{aligned} J(u_k)-J^*=O\left( \frac{1}{k}\right) . \end{aligned}$$

For the strongly convex objective function, it is known that the iterative sequence $\left\{ u_k \right\}$ converges linearly to the unique solution. However, it is not possible to show convergence for the iterative sequence $\left\{ u_k \right\}$ for the general convex case. Here, thanks to Assumptions A1–A5, we are able to prove that the iterative sequence $\left\{ u_k \right\}$ generated by the GPM converges strongly to an optimal control. Moreover, the convergence rate is established, depending on the constants $\theta$ appearing in Assumption A5.

The following estimate will be used repeatedly in our convergence analysis.

### Proposition 3.1

Let Assumptions A1–A4 be satisfied, let $u^*$ be a solution of (1.1)–(1.3) such that Assumption A5 is fulfilled with some $\theta >0$ and $\beta >0$. Then for all $k \in {\mathbb {N}},$ the following estimate holds

3.4 $$\begin{aligned} \Vert u_{k+1}-u^*\Vert ^2\le & {} \Vert u_{k}-u^*\Vert ^2 -\left( 1-\lambda _k L \right) \Vert u_{k+1}-u_k\Vert ^2\nonumber \\&-\,2 \lambda _{k} \beta \Vert u_{k+1}-u^*\Vert ^{2\theta +2}. \end{aligned}$$

### Proof

Since $u_{k+1}=P_{{\mathcal {U}}}(u_k-\lambda _k \nabla J(u_k))$, it follows from (2.5) that

3.5 $$\begin{aligned} \langle u_k-\lambda _k \nabla J(u_k)-u_{k+1}, u - u_{k+1}\rangle \le 0 \quad \forall u\in {\mathcal {U}}. \end{aligned}$$

Substituting $u=u^*\in {\mathcal {U}}$ into the latter inequality yields

$$\begin{aligned} \langle u_k-\lambda _k \nabla J(u_k)-u_{k+1}, u^*- u_{k+1}\rangle \le 0, \end{aligned}$$

or equivalently

$$\begin{aligned} \langle u_k-u_{k+1}, u^*- u_{k+1}\rangle \le \lambda _k \langle \nabla J(u_k), u^*- u_{k+1}\rangle . \end{aligned}$$

This implies that

3.6 $$\begin{aligned} \Vert u_{k+1}-u^*\Vert ^2= & {} \Vert u_{k}-u^*\Vert ^2+2\left\langle u_k-u^*, u_{k+1}-u_k\right\rangle +\Vert u_{k+1}-u_k\Vert ^2 \nonumber \\= & {} \Vert u_{k}-u^*\Vert ^2+2\left\langle u_{k+1}-u^*, u_{k+1}-u_k\right\rangle -\Vert u_{k+1}-u_k\Vert ^2 \nonumber \\\le & {} \Vert u_{k}-u^*\Vert ^2+2\lambda _k \langle \nabla J(u_k), u^*- u_{k+1}\rangle -\Vert u_{k+1}-u_k\Vert ^2 \nonumber \\= & {} \Vert u_{k}-u^*\Vert ^2\nonumber \\&-\,2\lambda _k \left[ \langle \nabla J(u_k), u_{k+1}-u^*\rangle +\frac{L}{2}\Vert u_{k+1}-u_k\Vert ^2 \right. \nonumber \\&\left. +\left( \frac{1}{2\lambda _k}-\frac{L}{2} \right) \Vert u_{k+1}-u_k\Vert ^2 \right] \nonumber \\= & {} \Vert u_{k}-u^*\Vert ^2 -\left( 1-\lambda _k L \right) \Vert u_{k+1}-u_k\Vert ^2\nonumber \\&-\,2\lambda _k \bigg [ \langle \nabla J(u_k), u_{k}-u^*\rangle +\langle \nabla J(u_k), u_{k+1}-u_k\rangle \nonumber \\&+\,\frac{L}{2}\Vert u_{k+1}-u_k\Vert ^2 \bigg ]. \end{aligned}$$

Since *J* has Lipschitz derivative, we have from Lemma 2.1 that

$$\begin{aligned} J(v)-J(u)-\left\langle \nabla J(u), v-u \right\rangle \le \frac{L}{2}\Vert v-u\Vert ^2 \quad \forall u,v \in {\mathcal {U}}. \end{aligned}$$

Substituting $u=u_k$ and $v=u_{k+1}$ into the last inequality yields

3.7 $$\begin{aligned} -\langle \nabla J(u_k), u_{k+1}-u_k \rangle - \frac{L}{2}\Vert u_{k+1}-u_k\Vert ^2 \le J(u_k)-J(u_{k+1}). \end{aligned}$$

Moreover, since *J* is convex, we obtain

3.8 $$\begin{aligned} -\langle \nabla J(u_k), u_{k}-u^* \rangle \le J(u^*)-J(u_k) \end{aligned}$$

Combining (3.6), (3.7) and (3.8) gives

3.9 $$\begin{aligned} \Vert u_{k+1}-u^*\Vert ^2\le & {} \Vert u_{k}-u^*\Vert ^2 -\left( 1-\lambda _k L \right) \Vert u_{k+1}-u_k\Vert ^2\nonumber \\&-\,2\lambda _k \left( J(u_{k+1}) -J(u^*)\right) . \end{aligned}$$

Using Assumption A5 we obtain

$$\begin{aligned} \Vert u_{k+1}-u^*\Vert ^2\le & {} \Vert u_{k}-u^*\Vert ^2 -\left( 1-\lambda _k L \right) \Vert u_{k+1}-u_k\Vert ^2\\&-\,2\lambda _k \beta \Vert u_{k+1}-u^*\Vert ^{2\theta +2}, \end{aligned}$$

which is (3.4). $$\square $$

We are now in the position to establish the strong convergence and the convergence rate of $\left\{ u_k \right\}$ to a solution.

### Theorem 3.2

Let Assumptions A1–A4 be satisfied, let $u^*$ be a solution of (1.1)–(1.3) such that Assumption A5 is fulfilled with some $\theta >0$. Let the sequence $\left\{ \lambda _{k} \right\}$ be chosen such that

$$\begin{aligned} 0 < \lambda _{\min } \le \lambda _{k} \le \frac{1}{L} \quad \forall k \in {\mathbb {N}}. \end{aligned}$$

Then we have

(i) $\Vert u_{k}-u^*\Vert ^2 \le \eta k^{-\frac{1}{\theta }},$ for all *k*, where $\eta >0$ is a constant;
(ii) The sequence $\{J(u_k)\}$ is monotonically decreasing. Moreover $\sum _{k=0}^{\infty } \left( J(u_k)\right. \left. -J(u^*)\right) < +\infty .$

### Proof

We first prove that $\{u_{k}\}$ converges strongly to $u^*$. From (3.4) and $0 < \lambda _{\min } \le \lambda _{k} \le \frac{1}{L}$, the sequence $\left\{ \Vert u_{k}-u^*\Vert \right\}$ is decreasing and bounded from below by 0, and therefore it converges. Moreover, since

3.10 $$\begin{aligned} 2 \lambda _{\min } \beta \Vert u_{k+1}-u^*\Vert ^{2\theta +2} \le \Vert u_{k}-u^*\Vert ^2- \Vert u_{k+1}-u^*\Vert ^2 \end{aligned}$$

we conclude that $\left\{ \Vert u_{k}-u^*\Vert \right\}$ converges to 0, which means $\{u_{k}\}$ converges strongly to $u^*$.

Now we can apply Lemma 2.5 for $s_k= \Vert u_{k}-u^*\Vert ^2, \alpha =\theta$ and $\delta _k= 2\lambda _{\min } \beta$ to obtain the convergence rate (*i*) for $\left\{ \Vert u_{k}-u^*\Vert \right\}$.

Substituting $u=u_k$ in (3.5) implies

3.11 $$\begin{aligned} \lambda _k\langle \nabla J(u_k),u_k-u_{k+1}\rangle \ge \Vert u_{k+1}-u_k\Vert ^2. \end{aligned}$$

Combining (3.7) and (3.11) we get

3.12 $$\begin{aligned} J(u_{k+1})-J(u_k)\le \left( \frac{L}{2}-\frac{1}{\lambda _k}\right) \Vert u_{k+1}-u_k\Vert ^2\le 0. \end{aligned}$$

Hence the sequence $\{J(u_k)\}$ is monotonically decreasing. Now from (3.9) and $0 < \lambda _{\min } \le \lambda _{k} \le \frac{1}{L}$ we have

$$\begin{aligned} 2\lambda _{\min } \left( J(u_{k}) -J(u^*)\right) \le \Vert u_{k-1}-u^*\Vert ^2 -\Vert u_{k}-u^*\Vert ^2 \quad \forall k \in {\mathbb {N}}. \end{aligned}$$

Summing this inequality from 0 to $i-1$ we obtain

$$\begin{aligned} \sum _{k=0}^{i-1} \left( J(u_{k}) -J(u^*)\right) \le \frac{1}{2\lambda _{\min }}\left( \Vert u_{0}-u^*\Vert ^2 -\Vert u_{i}-u^*\Vert ^2 \right) . \end{aligned}$$

Finally, taking the limit as $i \rightarrow \infty$, we obtain (*ii*). $\square$

### Remark 3.3

From (ii) in Theorem 3.2, we can conclude that $J(u_{k}) -J(u^*)=o(\frac{1}{k})$, which significantly improves the error estimate $J(u_{k}) -J(u^*)=O(\frac{1}{k})$ in (3.3).

The following example illustrates that the estimation (i) in Theorem 3.2 cannot be improved when $\lambda _k$ is bounded from below by a constant $\lambda _{\min }$.

### Example 3.4

Consider the following optimal control problem

3.13 $$\begin{aligned}&\text{ minimize } \qquad \int _0^T\sigma (t)u(t)dt \nonumber \\&\text{ subject } \text{ to } \qquad u(t)\in U:=[-1,1]^m, \end{aligned}$$

where $\sigma$ is any continuous function fulfilling Assumption B3. Then $\nabla J(u)(t)=\sigma (t)$ is independent of *u* and the optimal control is given by $u^*(t)=-sgn(\sigma (t))$. Starting the GPM with $u_0\equiv 0$ and $\lambda _k=\lambda$ for some $\lambda \in {\mathbb {R}}^+$ we get

$$\begin{aligned} u_k(t)={\left\{ \begin{array}{ll}1, &{} \text{ if } \quad -k\lambda \sigma (t)>1,\\ -k\lambda \sigma (t), &{} \text{ if } \quad -1\le -k\lambda \sigma (t) \le 1,\\ -1, &{} \text{ if } \quad -k\lambda \sigma (t)<-1.\end{array}\right. } \end{aligned}$$

In the special case $\sigma (t)=t^\theta$, we therefore have $u_k(t)=\max \{-1,-k\lambda t^\theta \}.$ This implies that for $k>\frac{1}{\lambda T^{\theta }}$, we have

$$\begin{aligned} \Vert u_k(t)-u^*(t)\Vert ^2= & {} \int _0^{(k\lambda )^{-\frac{1}{\theta }}}(1-k\lambda t^\theta )^2 dt\\= & {} (k\lambda )^{-\frac{1}{\theta }}\left( 1-\frac{2}{\theta +1}+ \frac{1}{2\theta +1}\right) \\= & {} Ck^{-\frac{1}{\theta }}. \end{aligned}$$

For the objective value we get

3.14 $$\begin{aligned} J(u_k)-J(u^*)=\left( \frac{1}{\theta +1}-\frac{1}{2\theta +1}\right) (k\lambda )^{-1-\frac{1}{\theta }}, \end{aligned}$$

which is stronger than (ii). It remains unknown whether in the general case the estimation (ii) can be improved to an estimation similar to (3.14).

Using the stronger Assumptions B1–B2 the convergence rate of the corresponding trajectories can be obtained as a corollary of Theorem 3.2 and [22, Lemma 2].

### Corollary 3.5

Let Assumptions B1, B2 and A4 be satisfied and let $(x^*,u^*)$ be a solution of (1.1)–(1.3) such that Assumption A5 is fulfilled with some $\theta >0$. Further suppose that $\lambda _k\in [\lambda _{\min },1/L] \subset (0,1/L]$. Then the sequence $\{x_{k}(t)\}$ of trajectories converges strongly to the solution $x^*$. Moreover, there exists a positive constant *C* such that for all *k* it holds,

$$\begin{aligned} \Vert x_{k}-\hat{x}\Vert _c \le C k^{-\frac{1}{2\theta }}, \end{aligned}$$

where $$\Vert x(\cdot )\Vert _c=\max _{t\in [0,T]}|x(t)|$$.

When the Lipschitz modulus *L* is difficult to estimate, one can consider the non-summable diminishing stepsizes as follow.

### Theorem 3.6

Let Assumptions A1–A4 be satisfied, let $u^*$ be a solution of (1.1)–(1.3) such that Assumption A5 is fulfilled with some $\theta >0$. Let the sequence $\left\{ \lambda _{k} \right\}$ be chosen such that

$$\begin{aligned} \lim _{k \rightarrow \infty } \lambda _{k} =0, \quad \sum _{k=0}^{\infty }\lambda _{k} = \infty . \end{aligned}$$

Then the sequence $\{u_{k}\}$ converges strongly to $u^*$. Moreover there exists $N>0$ such that for all $k \ge N$, it holds

(i) $$ \Vert u_{k}-u^*\Vert ^2 \le C\mu _k^{-\frac{1}{\theta }}$$
(ii) $$J(u_k)-J(u^*)=o\left( \frac{1}{\mu _k}\right) $$,

where $\mu _k:=\sum _{i=N}^{k-1}\lambda _i$ and *C* is a constant.

### Proof

Let $\beta >0$ be as in Proposition 3.1. Since $\lim _{k \rightarrow \infty } \lambda _{k} =0$, there exists $N>0$ such that for all $k\ge N$ we have $1-\lambda _k L > 0$ and $2\lambda _k\beta <1$. From (3.4) we have that $\left\{ \Vert u_{k}-u^*\Vert \right\}$ is decreasing, therefore it converges. Moreover

$$\begin{aligned} 2 \lambda _{k} \beta \Vert u_{k+1}-u^*\Vert ^{2\theta +2} \le \Vert u_{k}-u^*\Vert ^2- \Vert u_{k+1}-u^*\Vert ^2 \quad \forall k \ge N. \end{aligned}$$

Using Lemma 2.5 with $s_k=\Vert u_{k+N}-u^*\Vert ^2, \alpha =\theta$ and $\delta _k:=2\lambda _{k+N}\beta$ we get that there exists $\gamma >0$ such that

$$\begin{aligned} \Vert u_{k}-u^*\Vert ^{2}\le \left( \Vert u_N-u^*\Vert ^{-2\theta }+\gamma \sum _{i=N}^{k-1}\lambda _i\right) ^{-\frac{1}{\theta }}\quad \forall k\ge N, \end{aligned}$$

which shows (i).

From (3.9), we have

$$\begin{aligned} 2\lambda _{k}\left( J(u_{k+1})-J(u^*) \right) \le \Vert u_{k}-u^*\Vert ^2-\Vert u_{k+1}-u^*\Vert ^2 \quad \forall k \ge N. \end{aligned}$$

leading to

$$\begin{aligned} \sum _{k=N}^{\infty } \lambda _{k} \left( J(u_{k+1})-J(u^*) \right) < \infty . \end{aligned}$$

Applying Lemma 2.6 with $\alpha _k=\lambda _{N+k}$ and $s_k=J(u_{N+k})-J(u^*)$ we obtain (ii). $\square$

Using the same example as above we can again show that the estimation (i) cannot be improved.

### Example 3.7

Consider the problem (3.13) with $\sigma (t):=t^\theta$ again. As before we use GPM with $u_0\equiv 0$ but now with non-constant $\lambda _k$. Denoting $\mu _k:=\sum _{i=0}^{k-1}\lambda _i$ we get $u_k(t)=\max \{-1,-\mu _k t^\theta \}.$ Hence for *k* big enough such that $\mu _k>\frac{1}{T^\theta }$ we have

$$\begin{aligned} \Vert u_k(t)-u^*(t)\Vert ^2= & {} \int _0^{\mu _k^{-\frac{1}{\theta }}}(1-\mu _k t^\theta )^2 dt\\= & {} \mu _k^{-\frac{1}{\theta }}\left( 1-\frac{2}{\theta +1}+\frac{1}{2\theta +1}\right) \\= & {} C\mu _k^{-\frac{1}{\theta }} \end{aligned}$$

and

$$\begin{aligned} J(u_k)-J(u^*)=\left( \frac{1}{\theta +1}-\frac{1}{2\theta +1}\right) \mu _k^{-1-\frac{1}{\theta }}. \end{aligned}$$

Similar to Corollary 3.5 we obtain

### Corollary 3.8

Let Assumptions B1, B2 and A4 be satisfied and let $(x^*,u^*)$ be a solution of (1.1)–(1.3) such that Assumption A5 is fulfilled with some $\theta >0$. Further let the sequence $\left\{ \lambda _{k} \right\}$ be chosen such that

$$\begin{aligned} \lim _{k \rightarrow \infty } \lambda _{k} =0, \quad \sum _{k=0}^{\infty }\lambda _{k} = \infty . \end{aligned}$$

Then the sequence $\{x_{k}(t)\}$ of trajectories converges strongly to the solution $x^*$. Moreover, there exists a positive constant *C* such that for all *k* it holds,

$$\begin{aligned} \Vert x_{k}-\hat{x}\Vert _c \le C \mu _k^{-\frac{1}{2\theta }}. \end{aligned}$$

## Numerical illustrations

In this section, we present some numerical experiments for a class of optimal control problems with bang–bang solutions namely linear–quadratic problem, described as follow.

4.1 $$\begin{aligned} \begin{array}{ll} \text{ minimize } &{} \psi (x,u) \\ \text{ subject } \text{ to } &{} \dot{x}(t)=A(t)x(t)+B(t)u(t)+d(t), \quad t\in [0,T], \\ &{}u(t)\in U:=[-1,1]^m, \\ &{}x(0)=x_0, \end{array} \end{aligned}$$

where

$$\begin{aligned} \psi (x,u):= & {} \frac{1}{2}x(T)Qx(T)+q^\top x(T) +\int _0^T \left( \frac{1}{2}x(t)^\top W(t)x(t)\right. \\&\left. +\,x(t)^\top S(t)u(t) \right) dt. \end{aligned}$$

Here we use the classical Euler discretization where the error estimate can be found in [1, 2, 5]. We choose a natural number *N* and define the *mesh size* $h:=T/N$. Since the optimal control is assumed to be bang–bang, we identify the discretized control $u^N:=(u_0, u_1,\ldots ,u_{N-1})$ with its piece-wise constant extension:

$$\begin{aligned} u^N(t)=u_i \,\, \text {for} \,\, t\in \left[ t_i,t_{i+1}\right) , \, i=0,1,\ldots ,N-1. \end{aligned}$$

Moreover, we identify the discretized state $x^N:=(x_0, x_1,\ldots ,x_{N})$ and costate $p^N:=(p_0,p_1,\ldots ,p_N)$ with its piece-wise linear interpolations

$$\begin{aligned} x^N(t)=x_i+\frac{t-t_i}{h}\left( x_{i+1}-x_i \right) , \,\, \text {for} \,\, t\in \left[ t_i,t_{i+1}\right) , \, i=0,1,\ldots ,N-1 \end{aligned}$$

and

$$\begin{aligned} p^N(t)=p_i+\frac{t_i-t}{h}\left( p_{i-1}-p_i \right) , \,\, \text {for} \,\, t\in \left( t_{i-1},t_i\right] , \, i=N,N-1,\ldots ,1. \end{aligned}$$

The Euler discretization of (1.1) is given by 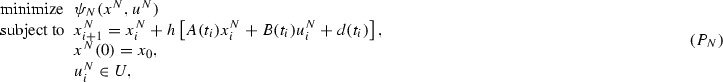 where $\psi _N$ is the cost function defined by

$$\begin{aligned} \psi _N(x^N,u^N) := \frac{1}{2} x_N^\top Qx_N+q^\top x_N +h \sum _{i=0}^{N-1}\left[ \frac{1}{2}x_i^T W(t_i)x_i+x_i^T S(t_i)u_i\right] . \end{aligned}$$

Observe that ($P_N$) is a quadratic optimization problem over a polyhedral convex set, where the gradient projection method converges linearly, see e.g., [30]. This means that for each *N*, there exists $\rho _N \in (0,1)$ such that

$$\begin{aligned} \left\| u^N_{k+1}-u^{N*}\right\| \le \rho _N \left\| u^N_{k}-u^{N*}\right\| , \quad \forall k \in {\mathbb {N}}. \end{aligned}$$

In the following examples, we will consider various values of *N* which suggest that

$$\begin{aligned} \lim _{N \rightarrow \infty } \rho _N =1. \end{aligned}$$

This will confirm the sublinear rate obtained in Theorem 3.2. The codes are implemented in Matlab. We perform all computations on a windows desktop with an Intel(R) Core(TM) i7-2600 CPU at 3.4 GHz and 8.00 GB of memory. Since $\nabla J$ is linear in *u*, one can roughly estimate its Lipschitz constant by $L=\Vert \nabla J(u_0)\Vert /\Vert u_0\Vert$. We choose starting control $u_0(t)=1 \, \forall t \in [0,T]$ and stepsize $\lambda _k =\lambda < 1/L$. The stopping condition is $\Vert u^N_k-u^N_{k-1}\Vert \le \epsilon$, where $\epsilon =10^{-10}$.

The following example is taken from [27].

### Example 4.1

4.2 $$\begin{aligned} \begin{array}{ll} \text{ minimize } &{} -by(1)+ \int _0^1\frac{1}{2}\left( x(t)\right) ^2dt \\ \text{ subject } \text{ to } &{} \dot{x}(t)=y(t), \quad x(0)=a \\ &{} \dot{y}(t)=u(t), \quad y(0)=1. \\ &{} u(t) \in [-1,1]. \end{array} \end{aligned}$$

Here, with appropriate values of *a* and *b*, there is a unique optimal solution $u^*$ with a switch from $-~1$ to 1 at time $\tau$, which is a solution of the equation

$$\begin{aligned} -5\tau ^4+24\tau ^3-(12a+36)\tau ^2+(24a+20)\tau +24b-12a-3=0. \end{aligned}$$

As in [27], we choose $a=1, b=0.1$, then $\tau =0.492487520$ is a simple zero of the switching function. Therefore, $\theta =1$ and the exact optimal control is

$$\begin{aligned} u^*(t)={\left\{ \begin{array}{ll} -1 &{} \text{ if } \, t\in [0,\tau ]\\ 1 &{} \text{ if } \, t\in (\tau , 1]. \end{array}\right. } \end{aligned}$$

**Fig. 1:**
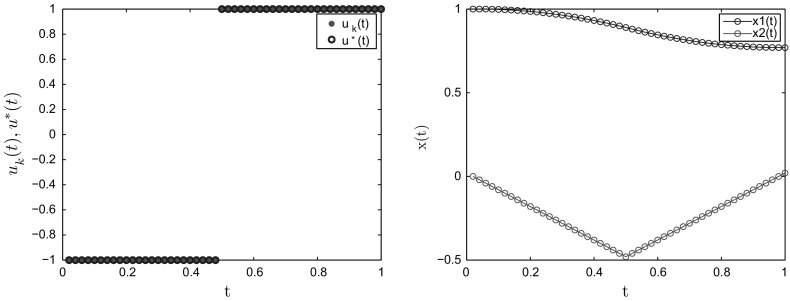
Optimal control (left) and optimal states (right) for $N=50$

The convergence results for Example 4.1 with some different values of *N* are reported in Table 1. We can see that when *N* increases, $\rho _N$ also increases and approaches 1. This means that we can only guarantee the sublinear convergence for the continuous problem. Figure 1 displays the optimal control and the optimal states when the discretized size $N=50$.

**Table 1:** Convergence rates for Example 4.1

N	10	20	50	100	200	500
$\rho _N$	0.7701	0.9181	0.9839	0.9902	0.9964	0.9976

The following second example is taken from [1, Example 6.1]

### Example 4.2

4.3 $$\begin{aligned} \begin{array}{ll} \text{ minimize } &{} \frac{1}{2} \left( \left( x_1(5)\right) ^2+\left( x_2(5)\right) ^2\right) \\ \text{ subject } \text{ to } &{} \dot{x_1}(t)=x_2(t), \\ &{} \dot{x_2}(t)=u(t),\quad \forall t\in [0,5]. \\ &{} x_1(0)=6, \quad x_2(0)=1,\\ &{} u(t) \in [-1,1]. \end{array} \end{aligned}$$

The exact optimal control is given by

$$\begin{aligned} u^*(t)={\left\{ \begin{array}{ll} 1 &{} \text{ if } \, t\in (\tau , 5]\\ -1 &{} \text{ if } \, t\in (0,\tau ], \end{array}\right. } \end{aligned}$$

where $$\tau = 3.5174292$$.

**Fig. 2:**
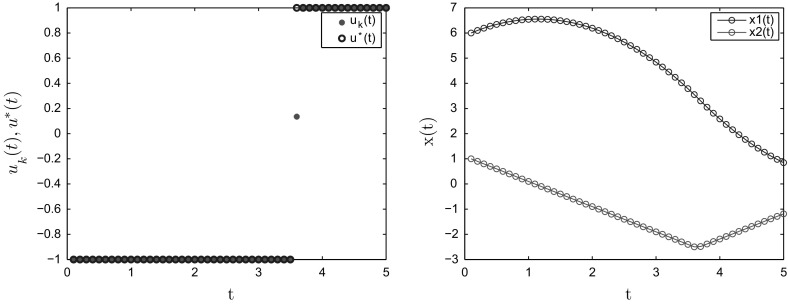
Optimal control (left) and optimal states (right) for Example 4.2 when $N=50$

The convergence results for Example 4.2 with some different values of *N* are reported in Table 2. Again, we see that when *N* increases, $\rho _N$ also increases and approaches 1. Figure 2 displays the optimal control and the optimal states for $N=50$.

**Table 2:** Convergence rates for Example 4.2

N	10	20	50	100	200	500
$\rho _N$	0.9625	0.9724	0.9905	0.9937	0.9943	0.9944

In the next example, we consider a problem in which Assumption A5 is satisfied for $\theta \not =1$ (see also [27, 29]).

### Example 4.3

Here we present experiments with a family of problems satisfying Assumption A5 with various values of $\theta$, given in [29]. Below, the time-interval is [0, 1], the dimension of the state is $n=\theta +1$ and the dynamics system depends on parameters $s_j$:

4.4 $$\begin{aligned} \begin{array}{ll} \text{ minimize } &{} x_1(1) \\ \text{ subject } \text{ to } &{} \dot{x_j}(t)=s_j x_{j+1}(t)+u(t), \quad j=1,\ldots ,\theta \\ &{} \dot{x}_{\theta +1}(t)=u(t), \\ &{} x(0)=0,\\ &{} u(t) \in [-1,1]. \end{array} \end{aligned}$$

**Table 3:** Convergence rates for Example 4.3

N	10	20	50	100	200	500
$$\theta =2$$						
$\rho _N$	0.9418	0.9686	0.9865	0.9962	0.9953	0.9947
$$\theta =3$$						
$\rho _N$	0.9245	0.9781	0.9936	0.9922	0.9968	0.9986

**Fig. 3:**
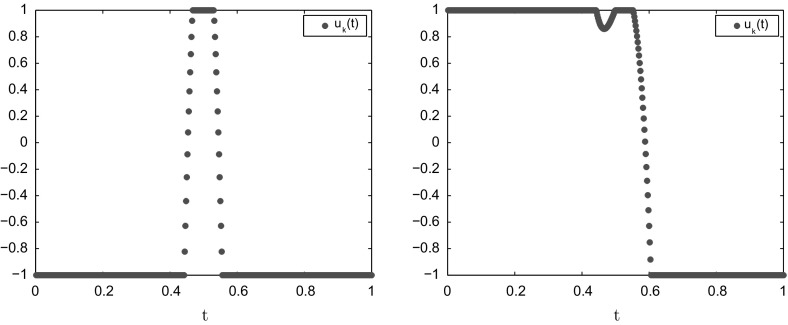
Approximate optimal controls after 1000 iterations when $\theta =2$ (left) and $\theta =3$ (right) for Example 4.3 with $N=500$

For any natural number $\theta$, the values of the parameters $s_j$ are chosen as

$$\begin{aligned} s_j:=-2(\theta -j+1) \quad j=1,\ldots ,\theta . \end{aligned}$$

Then Assumption A5 is satisfied with the constant $\theta$ [29] and exact optimal control is given by

$$\begin{aligned} u^*(t)={\left\{ \begin{array}{ll} 1 &{} \text{ if } \, t\in [0, 1/2]\\ -1 &{} \text{ if } \, t\in (1/2,1] \end{array}\right. } \end{aligned}$$

if $\theta$ is odd, and $u^*(t)=-1$ if $\theta$ is even. The convergence results for Example 4.3 when $\theta =2,3$ with some different values of *N* are reported in Table 3. Figure 3 displays the approximate optimal controls after 1000 iterations for $N=500$. It seems like the optimal control has $\theta$ switching points. This is to be expected since $\sigma ^*$ has a zero of order $\theta$ at 1 / 2.

## Concluding remarks

Note that the main results in Theorems 3.2 and 3.6 use Assumption A5 which is more general than just the bang–bang case. For example Assumption A5 is also satisfied in the strongly convex case, where even better convergence results are known. Further it would be interesting to see under what assumptions our results still apply in the case of singular arcs. This is challenging due to the fact that currently there is no condition similar to the bang–bang Assumption B3 that ensures Assumption A5 and therefore remains as a topic for future research.
